# Beneath the surface: the emerging concern of covert stroke in surgery

**DOI:** 10.1016/j.bjane.2025.844669

**Published:** 2025-08-21

**Authors:** Adrian W. Gelb, Bruna Bastiani, Cristiane Tavares

**Affiliations:** a University of California San Francisco, Department of Anesthesia & Perioperative Care, Center for Health Equity in Surgery and Anesthesia, San Francisco, California, USA; b Universidade Federal do Paraná (UFPR), Departamento de Anestesiologia, Curitiba, PR, Brazil; c Faculdade Evangélica Mackenzie do Paraná, Departamento de Pós-Graduação, Princípios de Cirurgia, Curitiba, PR, Brazil; d Faculdade de Medicina da Universidade de São Paulo (FMUSP), Instituto de Psiquiatria, Departamento de Anestesiologia, São Paulo, SP, Brazil

The incidence of stroke in patients undergoing non-cardiac, non-neurological surgery ranges from 0.1% to > 1%, with a reported mortality rate of 25%‒50%, substantially higher than that associated with non-perioperative strokes.^1^^,^^2^ These data, largely derived from retrospective studies,^1^ reflect variations in patient comorbidities and the nature of the surgical procedures. Perioperative stroke places a burden on healthcare systems, prolonging hospital stays, increasing ICU utilization, and often requiring discharge to chronic care facilities. These outcomes are devastating for patients and their families.^1^

The World Health Organization had defined stroke as a “focal or global neurologic deficit of cerebrovascular cause that persists beyond 24 hours or is interrupted by death within 24 hours”.^3^ According to the consensus statement from the Society for Neuroscience in Anesthesiology and Critical Care (SNACC), perioperative stroke includes ischemic or hemorrhagic cerebrovascular events occurring intraoperatively or within 30 days post-surgery.^4^ Overt perioperative strokes, those with clinical manifestations, are well characterized. Their etiology is primarily embolic rather than hypotensive, though intraoperative hypotension may exacerbate embolic injury. Risk factors such as chronic kidney disease, advanced age, previous transient ischemic attack or stroke, and atherosclerosis have been identified, yet many are non-modifiable and serve mainly to guide preoperative risk discussion.^1^, ^2^, ^3^

Covert Stroke, also known as Silent Brain Infarctions (SBI), are acute ischemic events detected exclusively by imaging, with no clinically apparent deficits.^1^^,^^5^ In 2013, the American Heart Association (AHA) modified the definition of stroke to include the extensive use of imaging technology: “Imaging or neuropathological evidence of CNS infarction, without a history of acute neurological dysfunction attributable to the lesion”.^6^ Their importance is underscored by their inclusion in the ICD-11 as silent cerebral infarction. However, it is noteworthy that the European Stroke Organization and World Stroke Organization do not endorse a definition of stroke that does not include symptoms.^6^

Population-based cohort studies report a wide prevalence (6%‒55%), reflecting variability in sample size, imaging protocols, and lack of standardized definitions.^7^ Frequency increases with age and vascular risk factors such as hypertension, carotid and coronary artery disease, hyperhomocysteinemia, oxidative stress, sleep apnea, and hypercoagulable states.^7^

There are numerous covert cerebrovascular lesions that are typically categorized as covert brain infarcts, White Matter Hyperintensities (WMHs), Cerebral Microbleeds (CMBs), and Perivascular Spaces (PVSs).^8^ Covert strokes, therefore, fall within this spectrum of Covert Cerebrovascular Diseases (CCD).^9^ CCD includes focal covert cortical infarctions, often due to atherothrombotic disease, and white matter diseases, such as lacunes, which result from intrinsic small vessel disease, microemboli, vasculopathy, amyloid deposition, and other related factors.^9^^,^^10^ The absence of clinical symptoms and only incidental imaging detection complicates understanding of the pathophysiology. The anatomical variability and the involvement of both large and small vessels suggest multiple mechanisms.^7^

Although patients with covert stroke may lack overt neurological deficits, these lesions have been linked to cognitive decline, increased risk of future strokes, and dementia. A meta-analysis carried out on the follow-up of more than 100,000 patients per year found that covert stroke was associated with an increased risk of stroke, with a crude RR of 2.94 (95% CI 2.24‒3.86),^5^ with an estimated annual stroke incidence of 3%, compared to less than 1% in those without. The risk of dementia is also significantly higher, 2%‒3% annually, versus 0.5% in those without occult cerebrovascular disease.^9^, ^10^, ^11^ These findings emphasize the potential clinical relevance of preoperative detection and management of covert stroke.

Two major prospective studies have recently investigated covert stroke in the postoperative setting. The NeuroVISION study enrolled 1,114 patients over age 65 undergoing elective non-cardiac, non-neurological surgery. Postoperative MRIs (days 2 and 9) and neurocognitive testing revealed a 7% incidence of covert stroke. These were associated with a higher risk of delirium, postoperative cognitive dysfunction, and transient ischemic attack.^12^ At one-year follow-up, 42% of patients with a covert stroke exhibited cognitive decline, compared to 29% in those without. The adjusted Odds Ratio was 1.98 (95% CI 1.22‒3.20), corresponding to a 13% absolute risk increase (p = 0.0055).^12^ In this trial*,* cognitive decline was defined as a reduction of two or more points on the Montreal Cognitive Assessment one year postoperatively compared to the preoperative baseline. Notably, and disappointingly, no additional risk factors were identified in that study, such as a previous history of stroke or transient ischemic attack, vascular disease, depression, anxiety, or type of surgery.^12^

The PRECISION study evaluated 934 patients over 60 years of age undergoing non-cardiac surgery, with postoperative MRI on day 7 and standardized assessments for delirium and cognitive outcomes.^13^ Of note, 66% of the cohort underwent neurosurgical procedures. The incidence of covert stroke was 11.9% (95% CI: 9.8%‒14.0%), well above the 7% found in the NeuroVISION study. In neurosurgical patients, the rate reached 16.3%, versus 3.4% among non-neurosurgical patients.^12^^,^^13^ A key distinction between the two studies is that the NeuroVISION excluded neurosurgical cases, whereas two-thirds of PRECISION’s cohort were neurosurgical, a reflection of recruitment challenges during the COVID-19 pandemic. The elevated incidence in PRECISION suggests a greater vulnerability and raises hypotheses regarding contributing factors such as brain manipulation, osmotic agents, or retractor use.

The very different patient selection in these two studies makes comparisons difficult. It also limits the ability to speculate about broader applicability to surgical populations and the extent to which they are confirmatory of each other. The incidence of covert stroke differs quite markedly in the non-neurosurgical populations, 7% vs. 3.4%, but even 3.4% is a surprising and disturbing finding.

Substantial gaps remain in understanding the timing and mechanisms underlying perioperative covert stroke, including whether these are the same or different from covert stroke unrelated to surgery. Neither study, unfortunately, was structured or powered to investigate risk factors or the contribution of intraoperative management to the incidence, including hemodynamic fluctuations and/or the generation of micro-emboli. Given that establishing a clear association between intraoperative hypotension and postoperative stroke remains challenging,^14^ identifying a link with covert stroke may be even more difficult and will require an explicit research agenda. In addition to identifying risk factors, diagnostic approaches will be crucial. Questions for consideration include the role of regionally specific neurological monitors such as regional cerebral oximetry, the use of Transcranial Doppler (TCD) in detecting microemboli; whether postoperative MRI should be routinely offered to patients with preoperative risk factors; whether the more readily available CT scan would be sufficient, or could frequent structured neurological evaluations, such as the modified NIH stroke scale, improve early detection of subtle deficits.^15^ Such unanswered questions highlight the very urgent need for appropriately powered prospective studies that clarify perioperative vulnerability and guide strategies within anesthetic and surgical practice.

In conclusion, covert stroke likely represents just the tip of a broader, underrecognized perioperative phenomenon. Its frequent underdiagnosis, stemming from the absence of overt symptoms and limited clinical suspicion, renders it a silent but impactful contributor to postoperative cognitive decline. This scenario calls for targeted, suitably powered research but also heightened awareness of covert stroke not only from anesthesiologists and surgeons but also from radiologists, ward physicians, ICU teams, and nursing staff involved in perioperative care. Interdisciplinary collaboration and systematic identification of at-risk patients are essential, at least initially for risk discussion. As evidence evolves, the development of robust, integrated guidelines for screening, diagnosis, and perioperative management will be key to reducing long-term neurological morbidity. Beneath the surface, the emerging concern of covert stroke in surgery demands collective recognition and deliberate action.

## Authors' contributions

All 3 authors contributed equally to the concept, literature review, and manuscript writing. All authors approved the final manuscript.

## Conflicts of interest

AWG is a past president of the World Federation of Societies of Anesthesiologists, and has a consulting relationship with Medtronic, and Haisco Pharma; all unrelated to this document.
